# Hidden bronchodilator responsiveness revealed after bronchoscopic removal of a tracheal lipoma: a case report

**DOI:** 10.3389/fmed.2026.1931137

**Published:** 2026-09-10

**Authors:** Yuhua Piao, Zhiguang Wang, Zhongjian An, Hongmei Piao

**Affiliations:** Department of Respiratory and Critical Care Medicine, The Affiliated Hospital of Yanbian University (Yanbian Hospital), Yanji, China

**Keywords:** airflow limitation, bronchodilator responsiveness, central airway obstruction, rigid bronchoscopy, tracheal lipoma

## Abstract

**Background:**

Tracheal lipoma is an exceptionally rare benign airway tumor that may present with non-specific respiratory symptoms and mimic common obstructive airway diseases, resulting in delayed diagnosis and inappropriate treatment. We report a case of tracheal lipoma presenting as persistent wheeze and apparently non-reversible airflow obstruction, in which significant bronchodilator responsiveness became evident only after bronchoscopic intervention.

**Case Presentation:**

A 72-year-old lifelong non-smoker presented with a one-month history of progressive wheeze and exertional dyspnea that was refractory to standard inhaled therapy. Pulmonary function testing demonstrated severe obstructive ventilatory impairment without bronchodilator reversibility. Contrast-enhanced chest computed tomography revealed a well-circumscribed, low-attenuation, non-enhancing intraluminal lesion in the distal trachea, raising suspicion for a benign adipose tumor. Rigid bronchoscopy identified a broad-based tracheal mass occupying more than 70% of the airway lumen. Complete endoscopic resection was successfully performed using electrocautery snare excision combined with carbon dioxide cryotherapy. Histopathological examination confirmed tracheal lipoma.

**Results:**

Following intervention, respiratory symptoms resolved rapidly, and airway patency was restored without complications. Pulmonary function improved substantially, with forced expiratory volume in 1 s (FEV_1_) increasing from 0.81 L to 1.49 L after bronchodilator administration. Notably, a previously negative bronchodilator test became markedly positive after tumor removal, obstructive airway physiology was relieved and BDR was unmasked.

**Conclusion:**

Bronchoscopic exploration shall be considered in patients presenting with persistent wheezing and poor response to routine treatment. Low-attenuation, non-enhancing tracheal lesions on CT may provide an important diagnostic clue. Rigid bronchoscopic intervention combined with electrocautery and cryotherapy is an effective minimally invasive treatment. This case highlights that fixed central airway obstruction can conceal underlying bronchodilator responsiveness, and relief of the obstruction may reveal clinically significant reversible airflow limitation.

## Introduction

Primary tracheal tumors are exceptionally rare, accounting for less than 1% of all neoplasms and occurring at an annual incidence of approximately 0.2 per 100,000 population (1, 2). Nearly 90% of primary tracheal tumors are malignant, predominantly squamous cell carcinoma and adenoid cystic carcinoma, whereas benign lesions comprise only a small proportion and include hamartoma, chondroma, lipoma, papilloma, and leiomyoma (2, 3). Tracheal lipoma is an exceedingly uncommon benign tumor arising from mature adipose tissue within the tracheal wall and represents less than 0.5% of all pulmonary tumors. Owing to its slow growth and non-specific clinical manifestations, diagnosis is frequently delayed.

Patients commonly present with cough, wheeze, exertional dyspnoea, or recurrent respiratory infections, symptoms that closely resemble asthma or chronic obstructive pulmonary disease (COPD). Consequently, many patients receive prolonged empirical treatment before the underlying central airway lesion is recognized. In addition to diagnostic delay, fixed tracheal obstruction may substantially alter pulmonary function measurements and obscure coexisting distal airway abnormalities. However, the physiological interaction between fixed central airway obstruction and bronchodilator responsiveness has received little attention in the literature. In particular, whether a tracheal lesion can mask reversible airflow limitation and generate a false-negative bronchodilator response remains poorly understood.

Recent advances in interventional pulmonology have established bronchoscopic techniques as the preferred minimally invasive approach for the management of benign central airway tumors (4). Nevertheless, reports describing the physiological recovery of pulmonary function following bronchoscopic intervention remain limited. Here, we describe a patient with tracheal lipoma initially misdiagnosed as obstructive airway disease who developed a marked positive bronchodilator response after rigid bronchoscopic tumor removal. This case highlights that fixed central airway obstruction may conceal reversible distal airway dysfunction and emphasizes the importance of integrating imaging, pulmonary function testing, and bronchoscopic evaluation in patients with persistent wheeze that is refractory to conventional therapy.

## Case presentation

A 72-year-old woman (height 158 cm, weight 55 kg, BMI 22.0 kg/m^2^) was referred to our department with a 1-month history of progressive wheezing and exertional dyspnea (mMRC Grade 2; Modified Borg Dyspnea Scale score 4). Symptoms gradually worsened despite treatment at a local hospital. She denied fever, hemoptysis, chest pain, weight loss, or a history of foreignbody aspiration. Chest CT performed at the local hospital demonstrated a 1.3 cm intraluminal lesion in the distal trachea accompanied by bilateral mucus plugging. The lesion was initially interpreted as a possible foreign body or mucus impaction. After 7 days of oxygen therapy, antibiotics, bronchodilators, and expectorants, repeat CT demonstrated partial resolution of mucus plugging but persistence of the tracheal lesion. Spirometry revealed severe obstructive ventilatory impairment with a negative bronchodilator test. Because symptoms persisted, the patient was referred to our institution. The patient experienced a cerebral infarction 10 years previously, with no residual neurological deficits. She denied smoking, alcohol consumption, occupational dust exposure, biomass fuel exposure, drug allergy, and food allergy.

Admission physical examination: T 36.0 °C, P 80 beats/min, R 18 breaths/min, BP 105/75 mmHg. Oxygen saturation was 94% at rest and decreased to 88% on exertion. Mild cyanosis of the lips was noted, along with diminished bilateral breath sounds and inspiratory wheeze. Cardiovascular and abdominal examinations were unremarkable.

**FIGURE 1:**
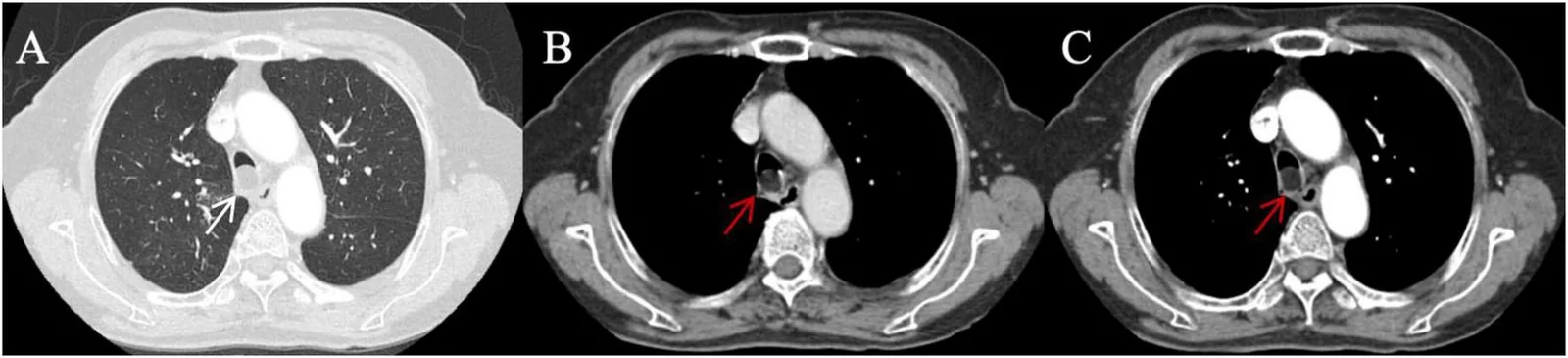
Enhanced chest CT of the tracheal tumor in the lower third: **(A)** non-contrast CT **(B)** venous-phase contrast CT **(C)** arterial-phase contrast CT.

Laboratory investigations, including complete blood count, inflammatory markers, serum biochemistry, and coagulation studies, were normal. Tumor markers, including carcinoembryonic antigen, cytokeratin19 fragment, neuron specific enolase, squamous cell carcinoma antigen, and progastrin releasing peptide, were within reference ranges. Preoperative spirometry (local hospital) demonstrated severe airflow obstruction with FEV_1_0.81 L (46.8% predicted), FVC 1.65 L (78.6% predicted), FEV1/FVC 48.76%; bronchodilator reversibility was negative (FEV1 rise < 12% and <200 mL following 400 mg salbutamol). Contrast enhanced chest CT demonstrated a well-defined low density intraluminal lesion measuring approximately 1.4-cm in the lower third of the trachea. The lesion exhibited homogeneous fat attenuation (approximately −40 HU) without appreciable contrast enhancement (Figure 1). Bilateral lower lobe mucus retention and mild distal bronchiectatic changes were also present.

Considering the persistence of the lesion despite medical treatment, the differential diagnosis included mucus impaction, foreign body, benign tracheal tumor such as hamartoma, lipoma, papilloma, and primary tracheal malignancy (Table 1). The characteristic fat attenuation on CT raised suspicion for a benign adipose tumor and prompted bronchoscopic evaluation.

**TABLE 1:** Differential diagnosis.

Diagnosis	Supporting features	Features against diagnosis
Mucus impaction	CT showed mucus plugs	Persistent mass after treatment
Foreign body aspiration	Endotracheal lesion	No aspiration history; stable appearance on serial imaging
Benign tracheal tumor	Well-circumscribed lesion; low attenuation	Rare disease
Primary tracheal malignancy	Airway obstruction symptoms	Fat density, no enhancement, benign bronchoscopic appearance

**FIGURE 2:**
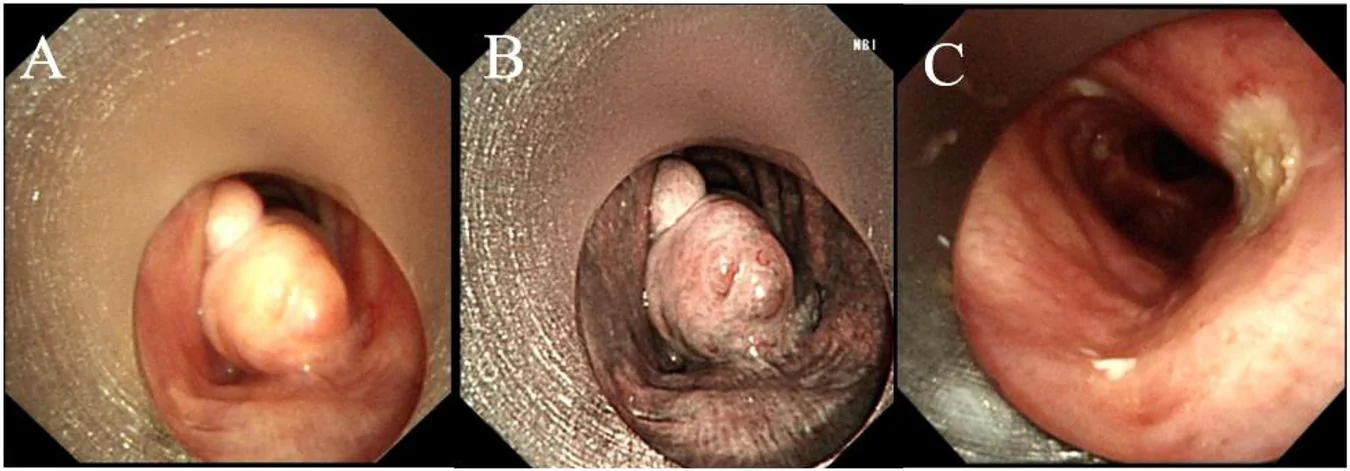
Rigid bronchoscopy of the tracheal tumor: **(A)** Before removal **(B)** NBI image **(C)** after removal.

Rigid bronchoscopy performed under general anesthesia revealed a smooth hemispherical broad based tumor arising from the distal tracheal wall and occupying more than 70% of the tracheal lumen. Narrow band imaging (NBI) demonstrated sparse superficial vascularity. Complete resection was performed using an electrocautery snare followed by carbon dioxide cryoextraction and cryotherapy of the tumor base. No significant bleeding or procedural complications occurred (Figure 2).

**FIGURE 3:**
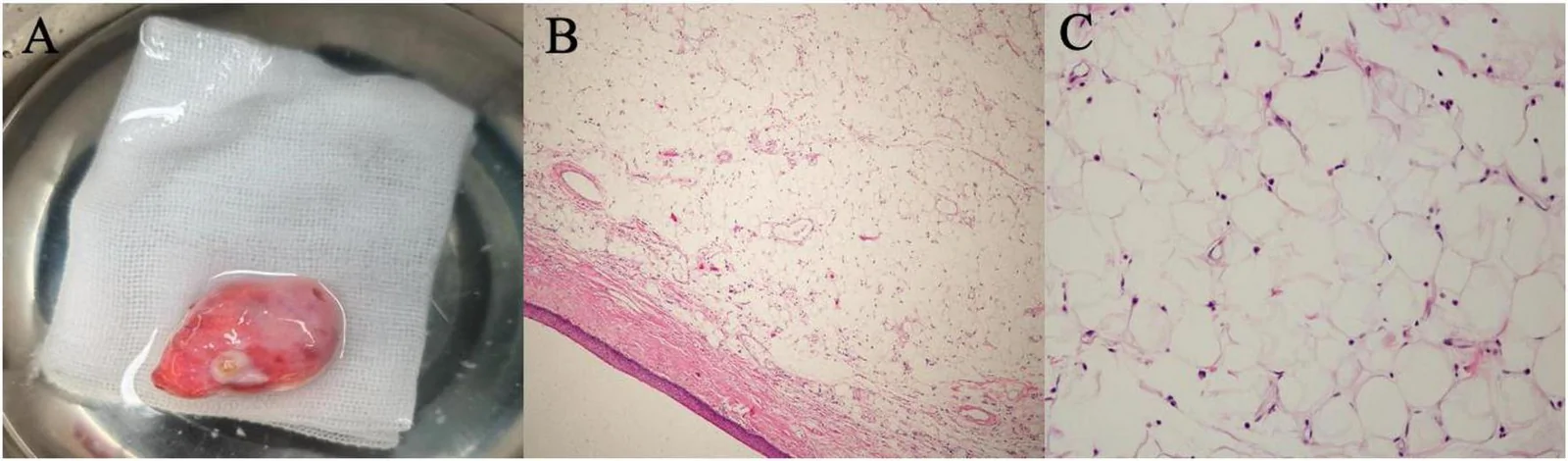
The bronchoscopic and microscopic view of the tracheal lipoma: **(A)** Tumor completely removed by electrocautery snare **(B)** Hematoxylin and Eosin staining (HE×100) **(C)** Hematoxylin and Eosin staining (HE×200).

Histopathological examination demonstrated mature adipocytes arranged in lobules separated by delicate fibrovascular septa beneath intact respiratory mucosa, confirming tracheal lipoma (Figure 3).

**FIGURE 4:**
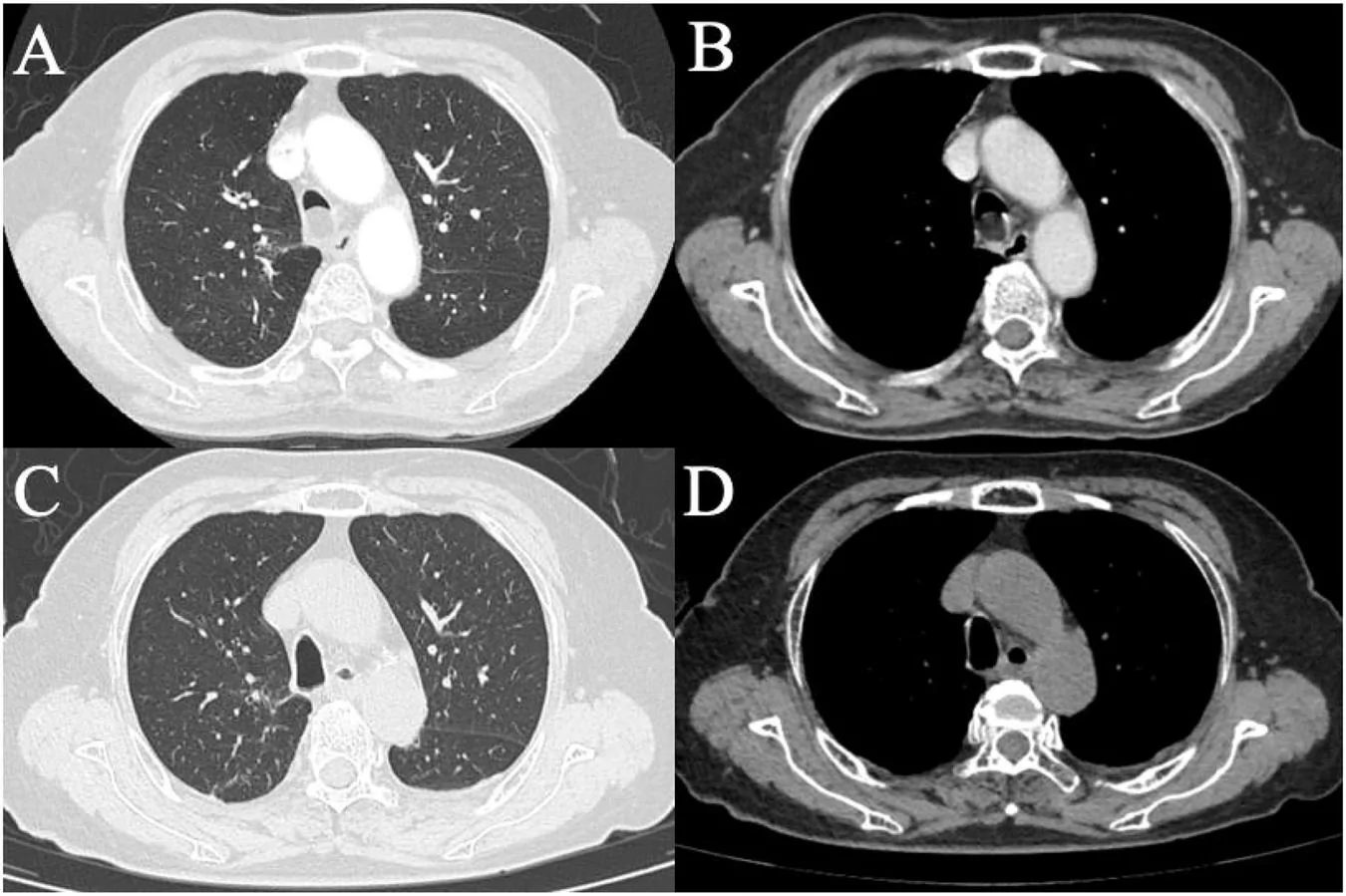
Chest CT images before and after treatment. **(A)** Preoperative contrast-enhanced chest CT (lung window setting) demonstrating a distal tracheal mass causing marked luminal narrowing. **(B)** Preoperative contrast-enhanced chest CT (mediastinal window, venous phase) showing a well-defined low-attenuation intraluminal lesion without appreciable enhancement. **(C)** Postoperative non-contrast chest CT (lung window setting) demonstrating restoration of tracheal patency following tumor removal. **(D)** Postoperative non-contrast chest CT (mediastinal window setting) confirming complete removal of the lesion without residual intraluminal abnormality.

Following treatment, wheezing and dyspnea improved rapidly. Postoperative CT confirmed near-complete removal of the lesion and restoration of tracheal patency (Figure 4) . Repeat spirometry demonstrated marked improvement in airflow limitation. FEV_1_ increased from 0.81 L to 1.01 L before bronchodilator administration and further increased to 1.49 L after bronchodilator inhalation. The bronchodilator response converted from negative to positive (Table 2). At 1 month follow up, the patient remained asymptomatic without evidence of recurrence.

**TABLE 2:** Pulmonary function tests before and after tumor debulking via rigid bronchoscopy.

Parameter	Pre-Debulking	Post-Debulking
FEV_1_ (L)	0.81	1.01
FVC (L)	1.65	1.81
FEV_1_/FVC (%)	48.76	56.08
PEF(L/s)	0.93	1.76
Bronchodilator response	Negative	Positive

## Clinical timeline

**Table:** 

Time	Clinical event
One month prior to admission	Progressive wheezing and exertional dyspnea (mMRC Grade 2; Modified Borg Dyspnea Scale score 4)
Local hospital assessment	CT demonstrated a distal tracheal mass with mucus plugging and severe airflow limitation
Over the subsequent 7 days	Oxygen, antibiotic, bronchodilator and mucolytic therapy were administered, yet no clinical improvement was observed
Repeat CT	Mucus plugging resolved, whilst the tracheal lesion remained unchanged
Admission to our hospital	Further CT imaging and rigid bronchoscopy were performed for characterization of the lesion
Interventional bronchoscopy	Complete endoscopic resection of the tracheal tumor was undertaken
Postoperative day 3	Marked alleviation of respiratory symptoms alongside recovery of pulmonary function
One-month follow-up	The patient remained entirely asymptomatic, with no clinical signs of tumor recurrence

## Diagnostic assessment and therapeutic intervention

The patient was initially treated as having obstructive airway disease because of progressive wheezing, dyspnea, and severe airflow limitation on spirometry. However, several findings were inconsistent with COPD or asthma, including the absence of smoking history, lack of response to bronchodilator therapy and persistence of a focal tracheal lesion on serial CT imaging. The characteristic fat attenuation of the lesion prompted bronchoscopic evaluation. Rigid bronchoscopy confirmed severe central airway obstruction caused by a distal tracheal tumor. Complete endoscopic removal was achieved using electrocautery snare excision combined with cryotherapy. Histopathological examination established the diagnosis of tracheal lipoma. Follow-up CT and spirometry demonstrated successful airway recanalization and substantial functional recovery. No recurrence or procedure-related complications were observed during follow-up.

## Discussion

Tracheal lipoma is an exceptionally rare benign tumor of the central airway and represents only a small fraction of primary tracheobronchial neoplasms (3, 5). Although histologically benign, progressive enlargement may result in substantial airway narrowing and clinically significant airflow limitation. Because of its indolent growth and nonspecific manifestations, diagnosis is frequently delayed until symptoms become severe. In the present case, a distal tracheal lipoma caused marked luminal obstruction and severe obstructive ventilatory impairment, initially mimicking chronic obstructive airway disease. Following bronchoscopic resection, airway patency was restored and pulmonary function improved dramatically. More importantly, a previously absent bronchodilator response became clearly positive after intervention. This observation provides additional insight into the physiological interaction between fixed central airway obstruction and bronchodilator responsiveness.

Previous studies have demonstrated that tracheobronchial lipomas most commonly occur in middle-aged and elderly patients and often present with chronic cough, wheezing, exertional dyspnea, recurrent pneumonia, or symptoms suggestive of asthma or COPD (6, 7). In the largest review to date, Muraoka et al. analyzed 64 cases of endobronchial lipoma and found that the majority of patients were diagnosed only after substantial airway obstruction had developed (8). More recent reviews have similarly emphasized that delayed diagnosis remains common because clinical manifestations overlap considerably with common obstructive airway disorders. Consistent with these observations, our patient had been treated empirically for presumed obstructive airway disease before the underlying tracheal lesion was recognized.

A particularly important clinical lesson from this case is the potential for central airway tumors to masquerade as asthma or COPD. Wheezing is traditionally regarded as a hallmark of lower airway obstruction. However, turbulent airflow through a narrowed tracheal lumen may generate auscultatory findings that are clinically indistinguishable from bronchospasm. Several published cases of tracheal or endobronchial lipoma have reported prolonged treatment for asthma before definitive diagnosis was established (2, 9, 10). These reports, together with the present case, highlight the importance of reconsidering the diagnosis when respiratory symptoms are disproportionate to conventional risk factors or fail to respond to standard therapy.

Radiological evaluation plays a pivotal role in the diagnosis of tracheal lipoma. Computed tomography typically demonstrates a well-circumscribed endoluminal lesion with homogeneous fat attenuation and little or no contrast enhancement (11, 12). In our patient, the lesion demonstrated classic radiological features of a benign adipose tumor. Nevertheless, it was initially interpreted as possible mucus impaction. The persistence of the lesion despite medical treatment prompted further investigation, ultimately leading to bronchoscopic confirmation. This experience underscores the importance of carefully assessing low-attenuation tracheal lesions and considering benign airway tumors in the differential diagnosis when imaging findings remain unchanged despite appropriate therapy.

Management strategies for tracheobronchial lipoma have evolved considerably over the past two decades. Surgical resection was considered the standard treatment for tracheobronchial lipomas, particularly when tumors extended beyond the airway wall or caused irreversible distal lung damage (13). Advances in interventional pulmonology have shifted treatment toward minimally invasive bronchoscopic techniques whenever technically feasible. Electrocautery, laser therapy, cryotherapy, argon plasma coagulation, and mechanical debulking have demonstrated excellent efficacy for benign endoluminal tumors while avoiding the morbidity associated with open airway surgery (14–17). In the present case, rigid bronchoscopy was selected because it provided optimal airway control, continuous ventilation, and superior instrument stability during tumor removal. Narrow-band imaging demonstrated limited vascularity, supporting the use of electrocautery snare resection. Subsequent cryotherapy was applied to the tumor base to minimize residual disease while reducing the risk of thermal injury to the tracheal cartilage. Complete recanalization of the airway was achieved without procedure-related complications, and the patient experienced rapid symptomatic improvement.

The most noteworthy finding in the present case was the marked change in bronchodilator responsiveness following airway recanalization. Before intervention, spirometry demonstrated severe airflow limitation and a negative bronchodilator test. After tumor removal, airflow parameters improved substantially and bronchodilator administration produced a significant increase in FEV1. Fixed central airway obstruction imposes a dominant flow-limiting segment within the respiratory system and may obscure the contribution of distal airway reversibility to overall airflow measurements. Following restoration of airway patency, the mechanical constraint imposed by the central lesion is removed, allowing underlying reversible airflow limitation to become detectable. Although causality cannot be definitively established from a single case, the present findings suggest that fixed central airway obstruction may mask clinically relevant bronchodilator responsiveness.

This report has several limitations. As a single-case observation, the proposed mechanism underlying the change in bronchodilator responsiveness remains speculative. In addition, follow-up duration was relatively short, precluding assessment of long-term functional outcomes and recurrence risk. Nevertheless, the clear temporal relationship between tumor removal, symptom resolution, restoration of airway patency, and improvement in pulmonary function provides valuable clinical insight.

## Patient perspective

Before the diagnosis, the patient believed that the symptoms were related to a common respiratory disease and became increasingly concerned as breathlessness progressively limited daily activities. Despite several medical treatments, the symptoms persisted and caused considerable anxiety. Following bronchoscopic removal of the tumor, the patient experienced immediate relief of dyspnea and was able to resume normal activities. The patient expressed satisfaction with the outcome and relief after finally receiving a definitive diagnosis.

## Conclusion

Tracheal lipoma should be considered in patients presenting with persistent wheezing and airflow limitation that are refractory to conventional therapy. Characteristic CT findings, combined with timely bronchoscopic evaluation, are critical for establishing the diagnosis. Rigid bronchoscopic intervention offers a safe and effective minimally invasive treatment option. Importantly, this case suggests that fixed central airway obstruction may conceal underlying bronchodilator responsiveness, and relief of the obstruction can reveal clinically significant reversible airflow limitation.

## Data Availability

The raw data supporting the conclusions of this article will be made available by the authors, without undue reservation.
